# Transparent conductive graphene textile fibers

**DOI:** 10.1038/srep09866

**Published:** 2015-05-08

**Authors:** A. I. S. Neves, T. H. Bointon, L. V. Melo, S. Russo, I. de Schrijver, M. F. Craciun, H. Alves

**Affiliations:** 1 INESC-MN and IN, Rua Alves Redol nº9, 1000-029 Lisboa, Portugal; 2 Centre for Graphene Science, College of Engineering, Mathematics & Physical Sciences, University of Exeter, EX4 4QL, United Kingdom; 3 Physics Department, IST, University of Lisbon, Av. Rovisco Pais 1, 1049-001 Lisboa, Portugal; 4 CenTexBel, Technologiepark-Zwijnaarde 7, 9052 Gent, Belgium; 5 Department of Chemistry, CICECO, University of Aveiro, 3810-193 Aveiro, Portugal

## Abstract

Transparent and flexible electrodes are widely used on a variety of substrates such as plastics and glass. Yet, to date, transparent electrodes on a textile substrate have not been explored. The exceptional electrical, mechanical and optical properties of monolayer graphene make it highly attractive as a transparent electrode for applications in wearable electronics. Here, we report the transfer of monolayer graphene, grown by chemical vapor deposition on copper foil, to fibers commonly used by the textile industry. The graphene-coated fibers have a sheet resistance as low as ~1 kΩ per square, an equivalent value to the one obtained by the same transfer process onto a Si substrate, with a reduction of only 2.3 per cent in optical transparency while keeping high stability under mechanical stress. With this approach, we successfully achieved the first example of a textile electrode, flexible and truly embedded in a yarn.

Electronic textiles offer a form of functional devices integrated in objects that are essential to human kind: clothes. Devices embedded in textiles would enable a form of electronics that is comfortable to wear, lightweight, easy to transport and imperceptible^1^. Such wearable electronics would find a wide range of applications from textile GPS and phones to biomedical monitoring, communication tools for the sensory impaired people, and personal security. Examples of textile embedded electronic devices have already arisen such as transistors^2^, electroluminescent devices^3^, supercapacitors^4^, or solar cells^5^. To achieve these results, a variety of materials compatible with textiles have been proposed, such as carbon nanotubes^6^, polymer nanocomposites^7^, oxide nanowires^8^, self-organized silicon nanowire network^9^, or ionic liquids^10^. In common to all devices is the need of a conducting transparent material to be used as electrodes, wiring, etc. Such material has to be flexible and produced at low temperature in a non-destructive processing deposition method, characteristics observed in carbon materials. Several materials have been proposed as transparent electrodes for wearable electronics. For example, the use of carbon nanotube-graphene hybrid fibers has enabled flexible electrodes with electrical conductivity of 10 S/cm or higher^6^,^11^. However, the use of chemical vapor deposition at high temperatures (as high as 1150 °C) is unsuitable for textile substrates. An alternative to direct growing is the use of graphene oxide (GO) which can produce conductive fibers at lower temperature^12^, through an electrical potential^13^, or by wet-spinning^14^. Furthermore, a nanocomposite comprised of chemically converted graphene and carbon nanotubes (CNT) has enabled to achieve a conductive layer with 240 Ω/sq at 86% transmittance on plastic substrates^15^, but in very caustic media with sulfuric acid and nitric acid for CNT dispersion and graphene production. Moreover, it is an exceptional challenge to assemble 2D microscopic chemically modified graphene sheets into macroscopic fibers due to their irregular size and shape and to unsteady layer stacking, thus only a few attempts have been made^16^. Nevertheless, none of these approaches results in the required transparency to fabricate completely built-in and concealed textile devices^17^

Monolayer graphene offers transparency, flexibility and mechanical strength combined with electrical conductivity^18^,^19^,^20^,^21^,^22^. In addition, its conductivity can be enhanced by chemical functionalization^23^,^24^,^25^ and its Fermi energy can be shifted by engineering the work function through chemical doping, and adjust the electrode with the carrier type needed^26^. Therefore, the use of graphene as electrode would minimize the contact resistance and reduce the operating voltages. Here, we demonstrate that conductive textile fibers, bendable and transparent can be obtained by wet transfer of monolayer graphene to a polypropylene and a polylactic acid biobased fiber. Polypropylene is one of the more commonly polymers used in textiles due to its specific weight, creating the lightest fiber yarn of all synthetic and natural fibers. The appropriate treatment of polypropylene leads to very different uses like diapers, underwear, or warm-weather clothing^27^,^28^. Polylactic acid is considered as the biopolymer par excellence to be applied in textile production due to its ability to be extracted from renewable natural resources by only one melt process from renewable natural resources, allied to its thermoplastic and mechanical properties^29^,^30^,^31^. The capacity of producing polypropylene and polylactic acid biobased conductive fibers maintaining the original aspect gives rise to completely embedded textile electronics.

## Results

### Coating of polypropylene fibers with graphene by transfer process

**Figure 1:**
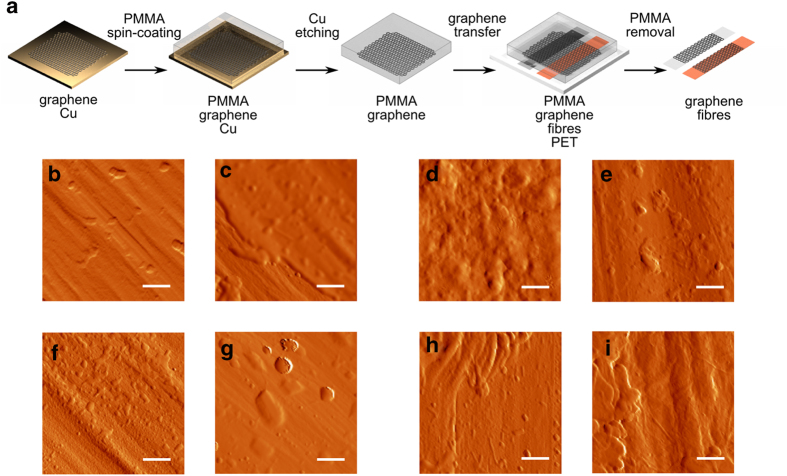
Graphene transfer process and AFM imaging. **(a)** General graphene transfer steps: CVD growth on copper; PMMA coating; copper etching; graphene transfer to the fibers and PMMA removal. AFM amplitude images (5 × 5 μm, scale bars are 1 μm) and roughness (Ra) taken at 45° with respect to the fiber length of **(b)** *PP (Ra* *=* *10.3* *nm)*; **(c)** *PP* *+* *G (Ra* *=* *8.3* *nm)*; **(d)** *PLA (Ra* *=* *25* *nm)*; **(e)** *PLA* *+* *G (Ra* *=* *12.3* *nm)*; **(f)** *PP UV (Ra* *=* *8.1* *nm)*; **(g)** *PP UV* *+* *G (Ra* *=* *7.7* *nm)*; **(h)** *PLA UV (Ra* *=* *20.1* *nm)*; and **(i)** *PLA UV* *+* *G (Ra* *=* *10.3* *nm)*. The UV treatment makes the fibers rougher but also more homogeneous, facilitating graphene adhesion. Image (c) shows that the graphene coverage in the untreated fibers is not complete.

Large-area monolayer graphene films were synthesized by chemical vapor deposition (CVD) on copper foil using methane as carbon source. After growth, a thin poly(methylmetacrylate) (PMMA) film was spin-coated on the graphene/Cu substrate to support mechanically the graphene layer during the following copper etching process. Graphene monolayer sheets were transferred to polypropylene fibers after copper etching, with following hot acetone cleaning to remove the PMMA layer. Fig. 1a illustrates the main steps used to coat the textile fibers with graphene. The fibers chosen as substrates presented a surface with roughness of 10 nm (determined from AFM measurements, see Fig. 1b) and two different types of fibers were used, monofilaments of polypropylene in the form of tape (PP) and biobased monofilaments of polylactic acid (PLA). After the PMMA layer was carefully removed, a fiber coated with a continuous graphene monolayer was obtained. The graphene sheet was in direct contact with the polymer fiber, without tears, conforming to the scaffold structure of the fiber. Fig. 1b-e shows the atomic force microscope (AFM) images of the fibers before and after the graphene transfer. A photo-sensitized oxidation process was also used on the fibers prior to the graphene deposition, employing ultraviolet-ozone (UVO) treatment. The UVO treatment promotes the elimination of solvent residues by exciting and dissociating the solvent molecules through absorption of short-wavelength ultraviolet radiation and their reaction with ozone. This leads to desorption of the contaminating molecules and to a more uniform and smooth polymer surface, and consequently a better adhesion of graphene (Fig. 1f-i). Even if the UVO treatment increased the general roughness, both in the PP and PLA fibers, we observed that the larger irregularities due to the polymerization process were diminished. As a result, the fiber surface acquired a more uniform and even structure. This gain in homogeneity upon the UVO treatment seems to also facilitate the electrostatic adhesion of the graphene sheet. In fact, only untreated fibers show some areas with small tears on the graphene sheet, which most likely result from loss of freestanding graphene during the PMMA removal process. These can be seen in the AFM phase signal which clearly presents two different types of interactions related to rigidity and adherence, which probably corresponds to sections covered with graphene and uncovered segments where the fiber surface is exposed (see Supplementary info). A similar adhesion behavior is observed in organic single crystals, where crystals with less than 1 μm thickness strongly stick through electrostatic forces to different substrates such as glass^32^, inorganic insulators (e.g. SiO_2_ or Al_2_O_3_^33^), metal films (e.g. Au, Pt or Ni^34^), flexible insulators such as polydimethyl siloxane (PDMS)^35^ and other organic crystals^36^ if the surface is sufficiently flat^37^.

**Figure 2:**
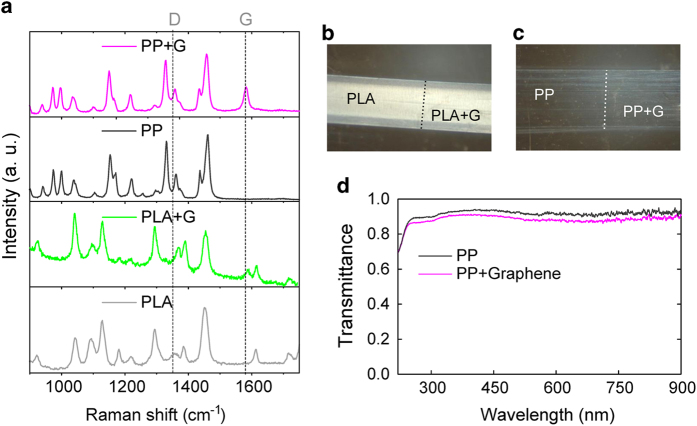
Optical response and Raman spectra. **(a)** Raman spectra for the bare *PP* (dark grey) and *PLA* (light grey) fibers and for the same fibers with graphene, *PP* *+* *G* (magenta) *PLA* *+* *G* (green), showing the characteristic graphene G band at *ca*. 1580 cm^−1^. The D band is not visible on the spectra, evidencing the quality of the monolayer graphene used. Photographs of the *PLA* **(b)** and *PP* **(c)** fibers centered at the edge of the transferred graphene sheet, with graphene on the right. Widths of the fibers are 1.2 mm for the PLA fiber and 1.6 mm for the PP fiber. **(d)** Transmittance spectra of the PP fiber coated with graphene (*PP* *+* *G*, magenta) and without graphene (*PP*, dark grey) measured with non-polarized light at normal incidence. Graphene coating results in a 3% decrease in the mean transmittance of the fiber.

A Raman spectroscopy study (using 532 nm wavelength excitation) was used to confirm the presence of graphene on the fibers. Fig. 2a shows the measured Raman spectra for the fibers with and without graphene. A clear G band (~1580 cm^−1^) can be observed for both the PLA and PP fibers coated with graphene, which are assigned to the E_2g_ vibrational mode of graphene. The 2D band (~2700 cm^−1^), also commonly used to identify graphene, overlaps with a region of intense and broad peaks intrinsic of the uncoated fibers. Nevertheless, the 2D band was observed on a control sample obtained by transferring a graphene film (grown under identical conditions) onto a SiO_2_/Si substrate (see Methods and Supplementary Info). The D band at ~1350 cm^−1^ 38 is commonly used to characterize the presence of defects in graphene, and, although the PP fiber have a considerable response in this region, this peak is absent in the PLA fiber, which has no background signal that can hinder the direct observation of this Raman peak. The presence of the D band is undoubtedly linked to density of defects, as seen, for example, in Ar^+^ bombarded graphene samples where increasing ion doses leads to increase in intensity and integrated area of the D band, which ultimate limits charge mobility^39^. The lack of observation of this band demonstrates that the transfer method used does not damage graphene. These Raman results indicate the presence of graphene on the fibers, yet, the fiber visual aspect remains unchanged (Fig. 2b and c). Comparing the transmittance of the transparent PP fibers, with and without graphene coating, in the visible region, the fiber with graphene presents a transmittance of 0.89 whereas the bare fiber has a transmittance of 0.92, which represents less than 3% loss in transparency (Fig. 2d). This result is consistent with the observed transmittance reduction of 2.3% of monolayer graphene with a further reduction of 2.3% for each extra layer of graphene^40^. The PLA fiber is opaque therefore a similar transmittance study was not viable.

### Conductive performance of graphene-coated fibers

**Figure 3:**
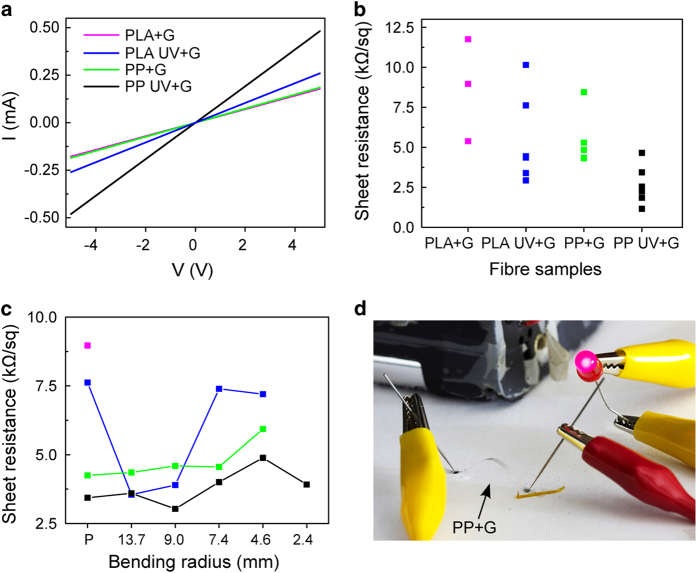
Sheet resistivity of the conducting fibers. (a) I-V curve of PP fiber with graphene (*PP* *+* *G*, green), PLA fiber with graphene (*PLA* *+* *G*, magenta), and the corresponding UV treated fibers with the graphene, *PP UV* *+* *G* (black) and *PLA UV* (blue). **(b)** Sheet resistivity of the samples. **(c)** Evolution of the calculated sheet resistivity of the samples with increasing bending. The *PLA* *+* *G* sample lost integrity with bending, while the other samples responded with a slight increase in sheet resistivity. **(d)** Photograph of an electric circuit with a LED light, closed with a bent and suspended transparent PP fiber coated with graphene. The visible graphite contacts were only used to define the channel length.

The electrical properties of the fibers coated with graphene were also investigated. Etching metal substrates and transferring conductive graphene films to flexible substrates such as polydimethylsiloxane (PDMS)^41^ or polyethylene terephthalate (PET)^42^ has successfully resulted in transparent electrodes. As-grown monolayer large-area CVD-grown graphene presents a sheet resistance (R_s_) > 1 kΩ/sq due to topological defects such as dislocations and grain boundaries^43^,^44^. These defects can disrupt the sp^2^ delocalization of π electrons in graphene and effectively scatter the charge carriers, forming highly resistive grain boundaries. Other defects such as wrinkles, folds, tears and cracks can also lead to a disrupted path of carrier transport, affecting the electrical resistivity^45^. However, we observe that even if the fibers have a rough surface morphology, the electrical conductance of graphene remains large, and the current *vs*. voltage characteristics is linear, as expected for ohmic conduction. Furthermore, for all the different fibers investigated here, the value of sheet resistance measured in air is typically lower than 12 kΩ/sq, with the best values reaching as low as 1 kΩ/sq (Fig. 3). These value are only slightly higher than the value observed for monolayer graphene transferred by the same method to Si substrates. Moreover we show that the sheet resistance decreases with the ultraviolet/ozone treatment, in accordance with the strongest uniformity and smoothness observed in the AFM images for treated fibers (Fig. 1). Flexible substrates usually require a smoothing layer, the most conventional method being sputtering and electron-beam evaporation of Al_2_O_3_^46^. Notably, in our study, no smoothing layer was necessary to reduced defects on surface flaws. Therefore, the transfer process to the fibers and their morphology do not seem to induce line defects in this two-dimensional crystal of carbon atoms.

### Flexibility of the conductive fibers

The effect of bending on the electrical resistance of the graphene-coated fibers was tested to analyze its suitability as a flexible electrode in textile electronics (Fig 3c). The foldability of the graphene films transferred to the tape fibers (thickness, 0.04 mm PP and 0.1 mm PLA) was tested by measuring the stability of the electrical resistance upon changing the bending radii. Most of the untreated PLA samples became insulating upon bending, even if only twisted with a 13.7 mm bending radius (approximate tensile strength of 0.4%). Untreated PP samples presented more stability upon bending and, for approximately one third of the samples, the sheet resistance showed little variation up to 7.4 mm bending radii, indicating that graphene presents a better adhesion to untreated PP fibers than to PLA fibers. However, the resistance of the UVO-treated fibers on both types of fibers show little variation up to a bending radius of 2.4 mm (approximate tensile strength of 2%). The limiting factor for the untreated samples seems to be the fiber itself, which is more fragile, and shreds upon bending (Fig. 3 in Supplementary info), creating breaks and discontinuities on the graphene sheet which lead to debonding of graphene. The UVO treatment not only smoothes the fiber surface and increases the adhesion of graphene, but it also seems to improve the mechanical strength of the fibers, in particular of the polylactic acid biobased fiber.

Finally to illustrate the high transparency, conductivity and flexibility of the graphene coated fibers, we show in Fig. 3d the operation of a light emitting diode (LED) in a circuit which contains the fiber as part of the conductive circuit wiring. The LED operates well even when the conductive fiber is bent.

## Discussion

Although most graphene derivative fibers have been presented as single components^12^,^13^,^14^,^16^, there are several examples of multilayer structures combining graphene with plastic substrates where also dissimilar materials with different thermo-mechanical properties are processed together. This gives rise to internal stress that has to be considered. Notably, the electrical stability upon bending of this first example of graphene-coated fibers is close to that of graphene films transferred to plastic substrates such as a PDMS/PET substrate, with an approximate tensile strength of 6.5%^41^, a value that already exhibited an excellent mechanical stability when compared to other materials used on flexible electronics^47^. The small difference is even more remarkable when the device geometry is considered. In the present case, the graphene coating is on the convex surface, more prone to failure, while on PDMS/PET substrate, graphene is in the neutral plane and can, therefore, be curled up to a tighter radius.

In summary, we have demonstrated that transparent monolayer graphene can coat textile fibers by wet transfer, forming a highly conductive and flexible thread with negligible aspect change. A surface treatment based on ultraviolet-ozone irradiation strongly promotes graphene adhesion to the fibers, leading to minor debonding upon on bending and high resistance stability. These first electrodes completely embedded in a textile fiber can pave the way for the development of devices for truly wearable, foldable, and transparent electronics.

## Methods

### Fibers fabrication

All the fibers have been fabricated by CenTexBel using a monofilament extrusion line.

### Graphene growth, transfer and surface treatments

Single-layer graphene was grown by chemical vapor deposition using a Moorfield nanoCVD-8G with a cold-wall reaction chamber, using methane as a carbon source and 0.025 mm thick 99.999% pure copper foil (Puratronic®, Alfa Aesar). The copper foils were annealed at 1035°C under H_2_ (0.4 sccm) for 10 min, the growth was performed under H_2_ (2 sccm) and CH_4_ (35 sccm) for 5 min, and then the chamber was rapidly cooled to room temperature for 10 min with a large Ar flow (50 sccm). The thickness and quality of the graphene film was first tested by transferring a CVD-grown graphene film onto a SiO_2_/Si substrate and examined by optical microscopy and Raman spectroscopy (see Supplementary Info). For the transfer process, a thin poly(methyl metracrylate) (PMMA 950 K A4) film was spin-coated on top of the graphene, and submitted to Ar plasma to etch any extra graphene on the backside. The copper was etched in an aqueous solution of ammonium persulfate 0.1 M, the floating graphene and PMMA sheet was washed several times with DI water. After this step, the graphene and PMMA sheet were transferred either to SiO_2_/Si or to the fibers (CenTexBel). In the case of the fibers, the graphene sheet was transferred by the scoop process with the fibers immobilized on a rigid PET support. Finally the PMMA was dissolved with acetone, leaving the graphene on the surface of SiO_2_/Si or of the fibers. The untreated fibers were used as received. UVO treatment of the fibers was performed using a UVO Cleaner® 144AX-220 (Jelight Company Inc.) for 11 min (6 min exposure time and 5 min exhaust).

### Microscopic characterization

The samples with and without graphene were characterized by AFM in multiple regions (Digital Instruments (Veeco/Bruker) Dimension 3100 with a Nanoscope IIIa controller) in tapping mode using commercial Olympus Si tips (OTESP), and by Raman spectroscopy (Horiba LabRam HR Evolution confocal Raman microscope with a 532 nm excitation laser and a 100x objective lens). The collected Raman radiation was dispersed with a 600 lines/mm grating and focused on a Peltier-cooled charge-couples device (CCD) detector allowing a spectral resolution of *ca*. 5 cm^−1^.

### Electric measurements

The electric conductivity was measured by a two-probe method using tungsten probes and a Keithley 237 source-measure unit. Graphite paste was used to draw the contacts and facilitate the measurements. The sheet resistance was calculated by subtracting the contact resistance and by taking into account the aspect ratio of the graphene section on each sample. To estimate the contact resistance we have used a method commonly used for graphene devices^48^,^49^, based on measuring the two-probe resistance in devices with different contact separation keeping the width of the channel constant. Since the two-probe resistance of a given sample is R_2p_ = R_G_ + 2R_C_, where R_G_ = ρL/W is the graphene resistance (ρ = graphene resistivity, L = channel length and W = channel width) and *R*_*c*_ is the contact resistance, we can estimate the contact resistance using samples with different lengths, L_1_ and L_2_ (W is the same). In this case, the contact resistance is given by R_c_ = (R_2p_^Dev1^-ρ_G_(L^Dev1^/W^Dev1^))/2 and ρ_G_ = (R_2p_^Dev1^-R_2p_^Dev2^)(L^Dev1^/W^Dev1^-L^Dev2^/W^Dev2^)^−1^. A contact resistance value of the order of 1 kΩ was found for our devices (Table S1 in Supplementary Info), which is in agreement with previous experiments^49^. The measurements were performed on the samples immobilized on flat glass plates and on cylinders with different radii (13.7, 9.0, 7.4, 4.6 and 2.4 mm).

### Optical measurements

Absorption spectra were measured with a scientific grade mini spectrometer (QE650000, Ocean Optics) with a resolution of 1.5 nm operating in the 200-950 nm range, coupled to a Deuterium Tungsten Halogen light source. Illumination and detection were done through optical fibers of 200 μm.

## Author Contributions

H. A. and M. F. C. conceived the work. A. I. S. N. prepared the samples and performed the experiments. T. H. B. prepared the conductive graphene. L. V. M. carried out the AFM imaging. I. S. developed the polyethylene fibers. A. I. S. N., H. A., S. R., and M. F. C. carried out the analysis. H. A. and M. F. C. co-supervised the work and H. A. wrote the manuscript.

## Additional Information

**How to cite this article**: Neves, A. I. S. *et al.* Transparent conductive graphene textile fibers. *Sci. Rep*. **5**, 9866; doi: 10.1038/srep09866 (2015).

## Supplementary Material

Supplementary Information
