# Supplementary material for: Transparent conductive graphene textile fibers
Source: Sci Rep. 2015 May 8;5:9866. doi: 10.1038/srep09866 (PMC4424659; doi:10.1038/srep09866)
Supplement: Supplementary Information [file srep09866-s1.pdf]

Supplementary Information for

Transparent conductive graphene textile fibers

A. I. S. Neves[1], T. H. Bointon[2], L. V. Melo[1,3], S. Russo[2], I. de Schrijver[4], M. F. Craciun[2] & H. Alves[1,5*]

[1] INESC-MN and IN, Rua Alves Redol nº9, 1000-029 Lisboa, Portugal.
[2] Centre for Graphene Science, College of Engineering, Mathematics & Physical Sciences, University of Exeter, EX4 4QL, United Kingdom.
[3] Physics Department, IST, University of Lisbon, Av. Rovisco Pais 1, 1049-001 Lisboa, Portugal.
[4] CenTexBel, Technologiepark-Zwijnaarde 7, 9052 Gent, Belgium.
[5] Department of Chemistry, CICECO, University of Aveiro, 3810-193 Aveiro, Portugal.
[*] email: halves@inesc-mn.pt; alves.helena@ua.pt.

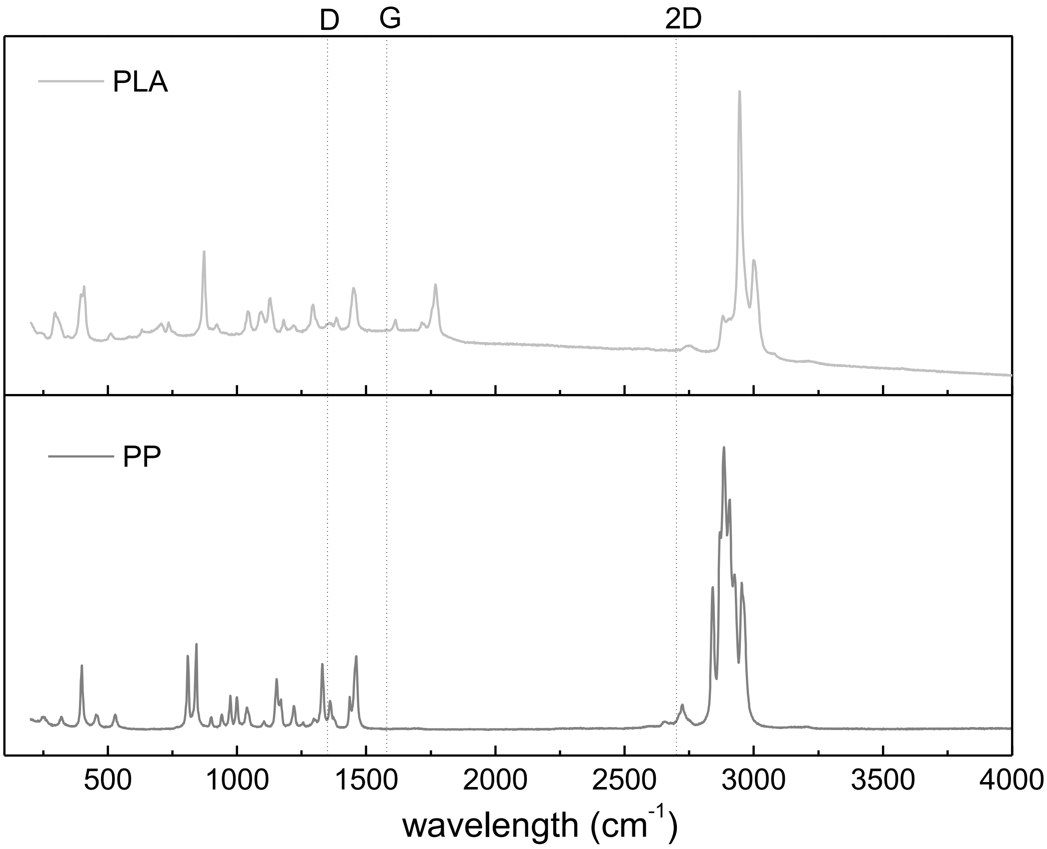

Figure S1. Raman spectra of the fibers without the graphene: PLA (top, light gray) and PP (bottom, dark gray) showing the whole wavelength range. Dotted vertical lines indicate the expected positions of graphene peaks D (1350 cm^{-1}), G (1580 cm^{-1}) and 2D (2700 cm^{-1}).

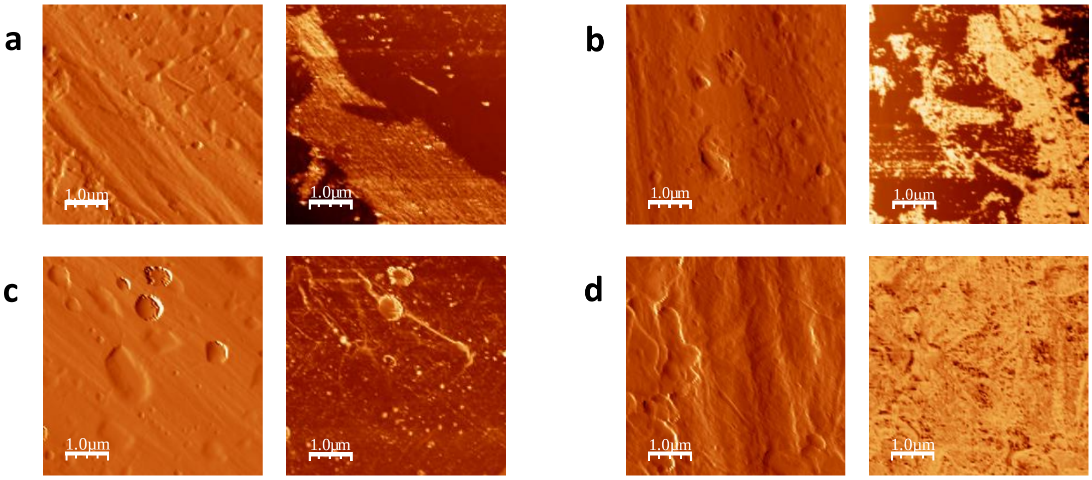

Figure S2. AFM amplitude and phase images for (a) PP+G, (b) PLA+G, (c) PP UV+G and (d) PLA UV+G. Two very different types of surface are visible in the phase AFM image on the non-UV-treated fibers (a) and (b), while a continuous coverage is achieved in the UV-treated fibers (c) and (d). Tapping mode amplitude is the tip oscillation amplitude while scanning, representing in fact a spatial derivative of the topography. The phase image represents the phase difference between the tip excitation signal and the tip oscillation and is sensitive to the tip-surface interaction.

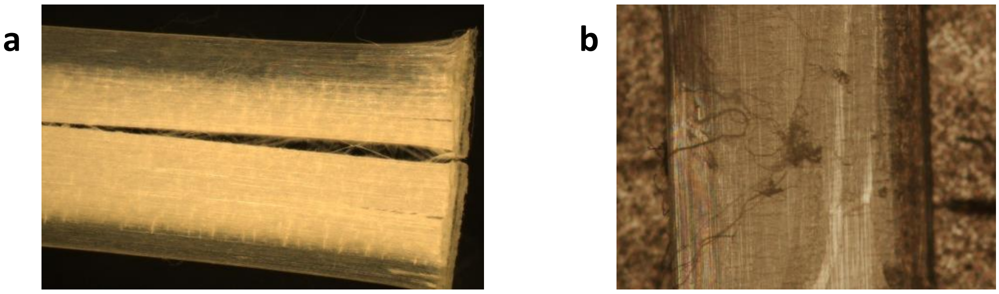

Figure S3. Detail of the damages on the PLA fiber: (a) split fiber; (b) loose microfibers on the surface.

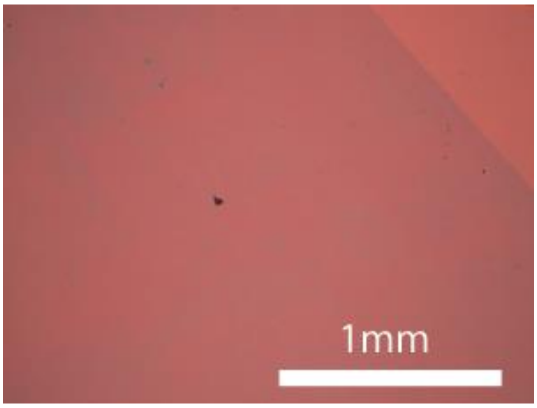

Figure S4. Optical microscope image of CVD graphene transferred onto an SiO_2/Si substrate via standard PMMA-assisted techniques. The edge of the film is shown to give contrast with the underlying substrate.

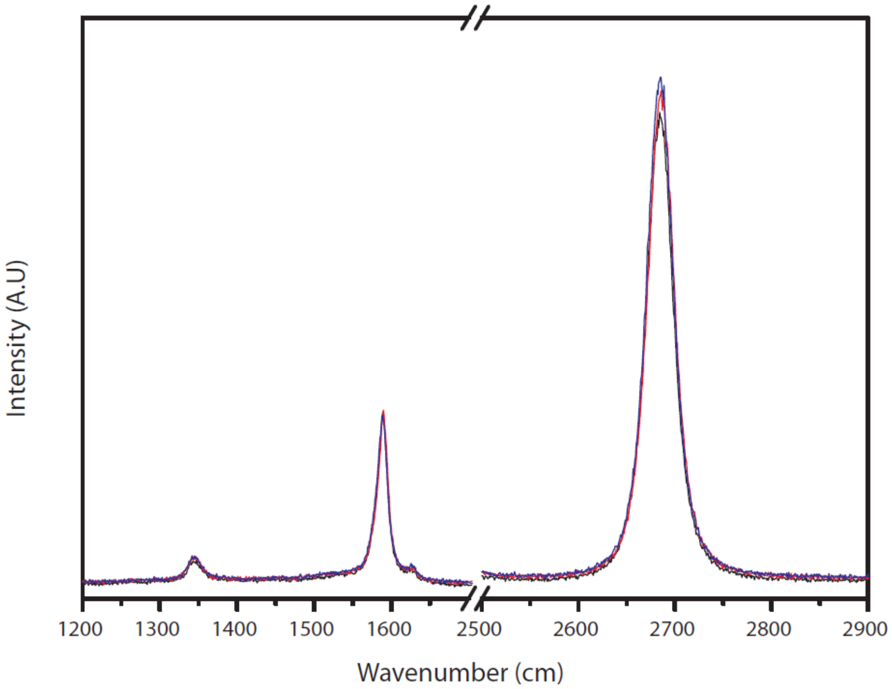

Figure S5. Three representative Raman spectra plotted for different regions of graphene transferred on SiO_2/Si.

Table S1. Examples of contact resistance estimation for the PP and PLA fibers. R_{2p} is the two probe resistance calculated from the I-V curves, L and W are the length and width of the fiber samples. $R_c=(R_{2p}^{Dev1}-\rho_G(L^{Dev1}/W^{Dev1}))/2$, where ρ_G is the graphene resistivity, $\rho_G=(R_{2p}^{Dev1}-R_{2p}^{Dev2})(L^{Dev1}/W^{Dev1}-L^{Dev2}/W^{Dev2})^{-1}$.

**Fiber type**	**Device no.**	**R_{2p} (Ω)**	**L (mm)**	**W (mm)**	**R_C (Ω)**
**PP**	Dev1	26502.87	93	16	1023.915
	Dev2	10199.51	31	16	
**PLA**	Dev1	18278.8082	45	12	720.8412
	Dev2	31000.1922	79	12	
